# Identification of an epithelial-mesenchymal transition related long non-coding RNA (LncRNA) signature in Glioma

**DOI:** 10.1080/21655979.2021.1951927

**Published:** 2021-07-21

**Authors:** Chuming Tao, Haitao Luo, Luyue Chen, Jingying Li, Xingen Zhu, Kai Huang

**Affiliations:** a Department of Neurosurgery, The Second Affiliated Hospital of Nanchang University, Nanchang, China; b Scientific Research Center, East China Institute of Digital Medical Engineering, Shangrao, China; c Department of Neurosurgery, Zhongshan Hospital Xiamen University, Xiamen, China; d Department of Comprehensive Intensive Care Unit, The Second Affiliated Hospital of Nanchang University, Nanchang, China; e Institute of Neuroscience, Nanchang University, Nanchang, China

**Keywords:** Epithelial-mesenchymal transition, glioma, long non-coding rna, prognostic signature

## Abstract

Epithelial–mesenchymal transition (EMT)-related long non-coding RNAs (lncRNAs) may be exploited as potential therapeutic targets in gliomas. However, the prognostic value of EMT-related lncRNAs in gliomas is unclear. We obtained lncRNAs from The Cancer Genome Atlas and constructed EMT-related lncRNA co-expression networks to identify EMT-related lncRNAs. The Chinese Glioma Genome Atlas (CGGA) was used for validation. Gene set enrichment and principal component analyses were used for functional annotation. The EMT–lncRNA co-expression networks were constructed. A real-time quantitative polymerase chain reaction assay was performed to validate the bioinformatics results. A nine-EMT-related lncRNAs (HAR1A, LINC00641, LINC00900, MIR210HG, MIR22HG, PVT1, SLC25A21-AS1, SNAI3-AS1, and SNHG18) signature was identified in patients with glioma. Patients in the low-risk group had a longer overall survival (OS) than those in the high-risk group (P < 0.0001). Additionally, patients in the high-risk group showed no deletion of chromosomal arms 1p and/or 19q, isocitrate dehydrogenase wild type, and higher World Health Organization grade. Moreover, the signature was identified as an independent factor and was significantly associated with OS (P = 0.041, hazard ratio = 1.806). These findings were further validated using the CGGA dataset. The low- and high-risk groups showed different EMT statuses based on principal component analysis. To study the regulatory function of lncRNAs, a lncRNA-mediated ceRNA network was constructed, which showed that complex interactions of lncRNA–miRNA–mRNA may be a potential cause of EMT progression in gliomas. This study showed that the nine-EMT-related lncRNA signature has a prognostic value in gliomas.

## Graphical Abstract

**Figure:**
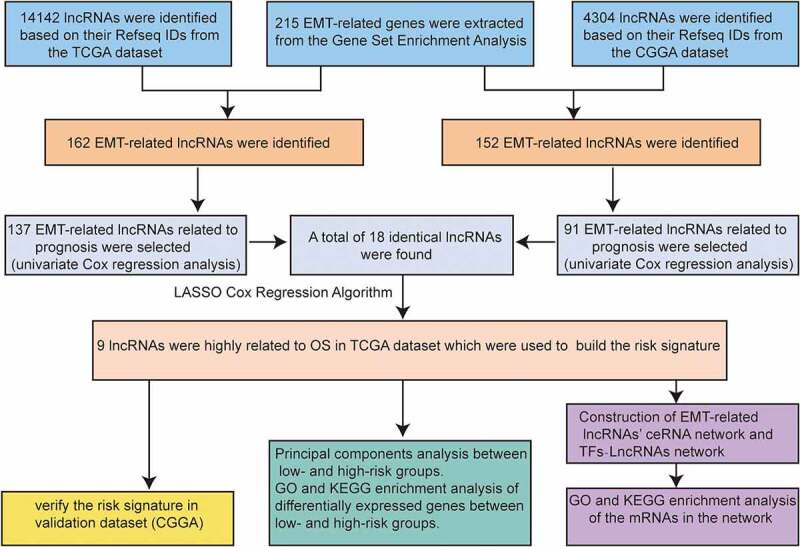

## Introduction

Gliomas are the most common type of malignant brain tumor in adults [1]. Glioblastoma (GBM; World Health Organization [WHO] grade IV) is the most invasive and aggressive glioma subtype with the highest mortality rate [2,3]. Epithelial–mesenchymal transition (EMT) is closely related to glioma malignancy [4,5], and lncRNAs are known to orchestrate multiple cellular processes by modulating EMT in various tumor cells [6]. Although the detailed mechanisms by which lncRNAs interact with EMT to promote glioma development and progression are not fully understood, their elucidation is critical for developing effective diagnostic and treatment modalities. The transdifferentiation of epithelial cells into motile mesenchymal cells plays an important role in development, wound healing, and stem cell behavior and contributes to the pathology of fibrosis and cancer progression [7]. Tumor cells undergoing EMT acquire resistance to apoptosis and antitumor drugs while disseminating to distant sites [8]. Thus, EMT is a hallmark of carcinogenesis, and strategies targeting EMT pathways have therapeutic potential for cancer treatment [9].

Long non-coding RNAs (lncRNAs) are RNA molecules with >200 bases that do not encode proteins [10,11] and have been shown to function as master regulators of gene expression in normal biological processes and in diseases such as cancer [12]. They are an important component of competing endogenous (ce) RNA networks and act as micro (mi) RNA sponges to regulate protein encoding and translation in cancer [13,14]. For example, upregulated TMPO antisense transcript 1 is a ceRNA that sponges miR-140-5p in gastric cancer cells, thereby alleviating the inhibition of SRY-box transcription factor 4, an EMT regulator [15]. Although lncRNAs related to EMT have been identified [16], there is a lack of data on their role in gliomas. The lncRNA–miRNA–mRNA ceRNA networks link the function of protein-coding mRNAs with that of non-coding RNAs such as miRNAs, lncRNAs, pseudogenic RNAs, and circular RNAs. We have found that LINC01133 inhibits gastric cancer progression and metastasis by acting as a ceRNA for miR-106a-3p to regulate adenomatous polyposis coli gene expression and the Wnt/β-catenin pathway [17]. SMAD5-AS1 targets miR-135b-5p to inhibit diffuse large B-cell lymphoma proliferation in the same pathway [18]. Therefore, deregulation of ceRNA networks may be an important factor leading to human diseases, including cancer [13].

In view of the important role of EMT and lncRNA in the occurrence and development of tumors, we aim at exploring the potential mechanisms of EMT-related lncRNAs in gliomas, we used data from The Cancer Genome Atlas (TCGA) to establish an EMT-related lncRNA signature for glioma that was subsequently validated using data from the Chinese Glioma Genome Atlas (CGGA). We also established a lncRNA-mediated ceRNA network to investigate the downstream targets and mechanisms of EMT-related lncRNAs.

## Material and methods

### Patients and datasets

Clinical information of patients with glioma was obtained from TCGA (https://cancergenome.nih.gov/) (13 March 2020) and the CGGA (http://www.cgga.org.cn/) (3 August 2019) websites; the two datasets were used for training and validation, respectively. The clinicopathological characteristics of the study population is shown in Table S1. Immune cell infiltration level data in tumors were downloaded from The Tumor Immune Estimation Resource (TIMER) online dataset data set (https://cistrome.shinyapps.io/timer/) [19]. This study was approved by the Ethics Committee of the Second Affiliated Hospital of Nanchang University.

### LncRNA profiling

The lncRNA profile was determined using an established mining method, by which genes were identified as protein coding or noncoding based on their Refseq IDs. Only long noncoding genes in the NetAffx annotation files were retained [20]. A total of 14,142 lncRNAs were obtained from TCGA dataset and 1,068 lncRNAs were obtained from the CGGA dataset. EMT-related genes were extracted using Gene Set Enrichment Analysis (http://www.broadinstitute.org/gsea/index.jsp) [21]. In total, 215 EMT-related genes were extracted. Ultimately, 162 EMT-related lncRNAs from TCGA and 152 EMT-related lncRNAs from the CGGA were retained by constructing EMT–lncRNA co-expression networks (cor = 0.6, P ≤ 0.0001).

### Establishment of a lncRNA signature

To establish a lncRNA signature for predicting survival, univariate Cox analysis was performed with the two datasets to determine the prognostic significance of lncRNAs. We screened lncRNAs with prognostic significance and identified 18 EMT-related lncRNAs that were closely associated with the survival of patients with glioma (P < 0.01). We then assigned a risk score using the least absolute shrinkage and selection operator (LASSO) Cox regression algorithm [22–24] and defined the inclusion criteria for nine lncRNAs and their constants, selecting the optimal penalty parameter β for a minimum 10-fold cross-validation in the training set. The risk score was calculated using the following formula:

Risk score = βgene1*exprgene1+ βgene2*exprgene2+ … … + βgenen× exprgenen [25]

where exprgenen is the expression level of a lncRNA. The low-risk and high-risk groups were distinguished based on the median risk score.

### Construction of ceRNA and TFs‐lncRNAs networks

The interaction between lncRNAs, miRNAs, and mRNAs in glioma was predicted based on overlapping miRNA seed sequence binding sites in lncRNAs and mRNAs. The interaction between the nine identified EMT-related lncRNAs and miRNAs was first predicted using the MiRcode database, which provides whole-transcriptome human miRNA target predictions based on the GENCODE annotation and includes >10,000 lncRNA genes. The miRTarBase, TargetScan, and miRDB databases were used to identify aberrantly expressed miRNA–mRNA pairs. We analyzed only target mRNAs that matched those in the databases. Finally, lncRNA–miRNA and miRNA–mRNA pairs were merged based on shared miRNAs into a ceRNA network of EMT-related lncRNAs. A list of 318 transcription factors (TFs) were obtained from the Cistrome Cancer dataset (http://www.cistrome.org/) [26]. The R package ‘limma’ was applied to identify differentially expressed TFs between low grade glioma (LGG) and GBM. All the networks were visualized using Cytoscape v3.7.0 software (The Cytoscape Consortium, San Diego, CA, USA).

### Use of reverse transcription quantitative polymerase chain reaction to validate bioinformatics results

We collected normal brain tissue (NBT) and glioma tissues, specifically, six NBTs, 10 lower-grade glioma (LGG) tissues, and nine GBM tissues from the Second Affiliated Hospital of Nanchang University from September 2018 to November 2020. Reverse transcription quantitative polymerase chain reaction (RT-qPCR) assay was conducted using a LightCycler® 480 real-time PCR system according to the manufacturer’s instructions. The expression levels of the nine selected lncRNAs were calculated using the 2-ΔΔCt method. The primer sequences for the nine selected lncRNAs are shown in Table S3.

### Statistical analysis

The expression of EMT-related lncRNAs between the two risk groups and their relationship with WHO grades were analyzed using one-way analysis of variance (ANOVA). To compare glioma characteristics with different clinical pathology risk scores, one-way ANOVA or a t-test was used to compare risk scores of patients grouped by clinical or molecular pathology characteristics. Univariate and multivariate Cox regression analyses and principal component analysis (PCA) were performed using R v3.6.3 and SPSS v22 software applications (SPSS Inc., Chicago, IL, USA). We performed differential gene expression analysis between the two risk groups, setting P < 0.05 and log2|fold change| > 1. GSEA was performed to investigate the functions of differentially expressed genes (DEGs) between the two groups. Univariate and multivariate Cox regression analyses were performed to determine the prognostic values of risk scores and various clinical and molecular pathological features including risk scores, age, sex, isocitrate dehydrogenase (IDH) status, and 1p/19q codeletion status. The Kaplan–Meier curve was generated using Prism v6 software (GraphPad, La Jolla, CA, USA). Receiver operating characteristic (ROC) curves were used to study the prediction efficiency of the risk signature, age, grade IDH-mutant status, and 1p/19q codeletion status. Gene Ontology (GO) and Kyoto Encyclopedia of Genes and Genomes (KEGG) pathway enrichment analyses were performed using the ‘clusterProfiler’ package of R software (Padj < 0.05 and Q < 0.05). Respectively, the 25 transcription factors (TFs) in the TFs‐lncRNAs network were then inputted into the ‘Metascape’ website for functional and pathway enrichment analysis [27]. GSEA was used for functional annotation of genes. A two-sided P value of <0.05 was considered significant for all statistical tests.

## Results

In the present study, we aimed to identify EMT-related lncRNAs in gliomas. We identified 18 prognostic EMT-related lncRNAs from TCGA and the CGGA datasets, and nine of them were used to construct a novel prognostic model. In addition, we performed ceRNA network, TFs‐lncRNAs network, and functional enrichment analysis to explore the potential mechanisms of EMT-related lncRNAs in gliomas.

### Identification of prognostic EMT-associated lncRNAs in glioma

We extracted 14,142 and 1,068 lncRNAs from TCGA and the CGGA datasets, respectively, and determined their expression profiles. A total of 215 EMT-related genes were extracted using GSEA (HALLMARK_EPITHELIAL_MESENCHYMAL_TRANSITION M5930, ANASTASSIOU_MULTICANCER_INVASIVENESS_SIGNATURE M2572). We identified EMT-related lncRNAs by constructing EMT-related lncRNA co-expression networks (cor = 0.6, P < 0.0001). Ultimately, 162 and 152 EMT-related lncRNAs were identified from TCGA and the CGGA, respectively.

### Establishment of an EMT-related lncRNA signature in glioma

**Figure 1.**
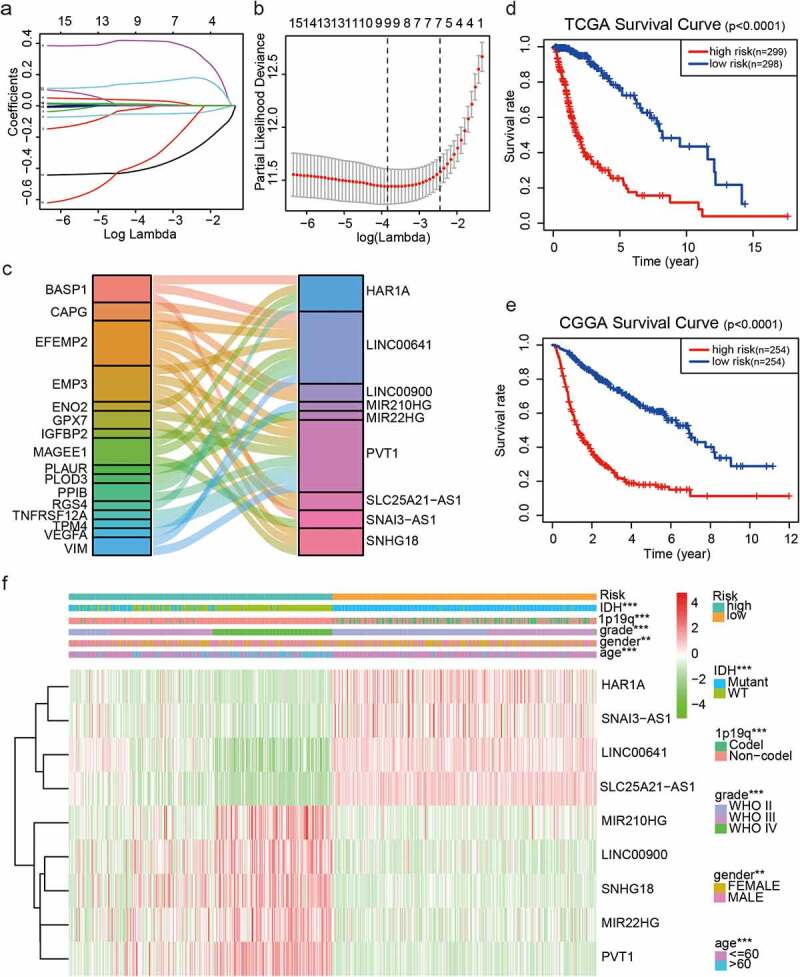
Risk signature consisting of nine EMT-related lncRNAs. (a, b) Inclusion criteria for the nine EMT-related lncRNAs and their constants; the optimal penalty parameter β has been selected for a minimum 10-fold cross-validation of the training set. (c) Seven EMT-related lncRNAs. (d, e) Kaplan–Meier curves for OS in patients in TCGA (high risk: n = 299, low risk: n = 298) and the CGGA (high risk: n = 254, low risk: n = 254) datasets assigned to high-risk and low-risk glioma groups based on the median risk score. (f) Heatmap of the nine EMT-related lncRNAs in low-risk and high-risk glioma. The distribution of clinicopathological features is compared between the low-risk and high-risk groups. ns P > 0.05, * P < 0.05, ** P < 0.01, and *** P < 0.001

To identify prognostic lncRNAs, expression data for each lncRNA in TCGA and the CGGA were analyzed using a univariate Cox proportional hazards regression model (P < 0.01). We selected 137 and 91 lncRNAs related to patient prognosis in glioma from TCGA and the CGGA, respectively. We screened lncRNAs with prognostic significance in both datasets and identified 18 lncRNAs that were significantly associated with the overall survival (OS) of patients with GBM (P < 0.01). LASSO Cox regression was used to identify the optimal prognostic lncRNAs in TCGA dataset and establish the risk signature. Nine of the 18 candidate lncRNAs retained their prognostic significance and were, thus, selected as independent prognostic markers (Figure 1a–c). Five EMT-related lncRNAs (LINC00900, MIR210HG, MIR22HG, PVT1, and SNHG18) were found to be high-risk lncRNAs (hazard ratio [HR] >1), and four EMT-related lncRNAs (HAR1A, LINC00641, SLC25A21-AS1, and SNAI3-AS1) were found to be protective lncRNAs (HR <1; Table 1). Based on the median risk score, we divided patients with glioma into the low-risk and high-risk groups (Table S2); OS was longer in the former group than in the latter group (P < 0.0001; Figure 1d). Next, we used the CGGA (n = 508) dataset as a validation dataset to calculate the risk scores. We found a significant difference in OS between the two risk groups (P < 0.0001; Figure 1e). The risk score was then applied to construct a heat map, which indicated that nine EMT-related lncRNAs were differentially expressed between the high-risk and low-risk groups (Figure 1f). WHO grade (P < 0.001), age (P < 0.05), IDH status (P < 0.001), 1p/19q codeletion status (P < 0.001), and sex (P < 0.001) were also significantly different between the two groups (Figure 1f). These results showed that the nine EMT-related lncRNAs and our risk models had good prognostic value in gliomas.

**Table 1.** The 9 EMT-related lncRNAs identified from the lasso cox regression in TCGA dataset

Symbol	HR	Low95	High95	P value	βValue
HAR1A	0.26	0.20	0.36	5.49E-18	−0.015
LINC00641	0.37	0.32	0.43	7.35E-38	−0.053
LINC00900	6.35	4.70	8.59	2.67E-33	0.417
MIR210HG	2.20	1.86	2.61	7.51E-20	0.036
MIR22HG	2.33	1.96	2.77	5.13E-22	0.007
PVT1	4.87	3.82	6.20	9.08E-38	0.146
SLC25A21-AS1	0.25	0.20	0.31	8.65E-39	−0.410
SNAI3-AS1	0.12	0.07	0.21	1.54E-15	−0.366
SNHG18	2.66	2.30	3.06	1.79E-41	0.012

### Validation of the 9-lncRNA prognostic risk signature

**Figure 2.**
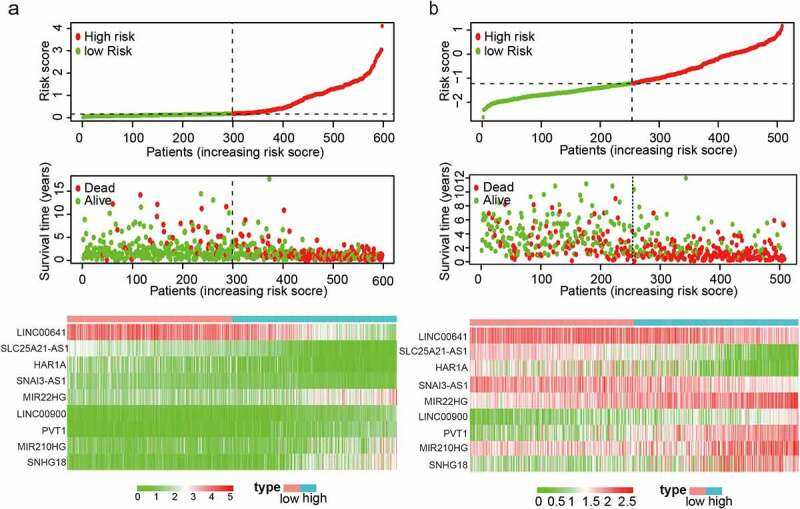
Distribution of risk scores, OS, and gene expression in TCGA and the CGGA datasets. (a, b) Distribution of risk scores and OS. (c) Heat map of the expression of nine EMT-related lncRNAs in low- and high-risk groups. Rows and columns show lncRNAs and patients, respectively

We ranked the risk scores of patients in TCGA training set, analyzed their distribution, and plotted their survival status (Figure 2a). The heatmap revealed differences in the expression patterns of the prognostic lncRNAs between the low-risk and high-risk groups. The expression of four lncRNAs was upregulated, whereas that of five risk-associated lncRNAs was downregulated in patients with low-risk scores; the opposite trend was observed in patients with high-risk scores. These results were confirmed using the CGGA validation set (Figure 2b). There are certain differences in the heatmaps of the two data sets of TCGA and CGGA. They are inconsistent in data value and standardization methods, but they will not affect the trend of the results.

### Expression of nine EMT-related lncRNAs and their relationship with patient prognosis

**Figure 3.**
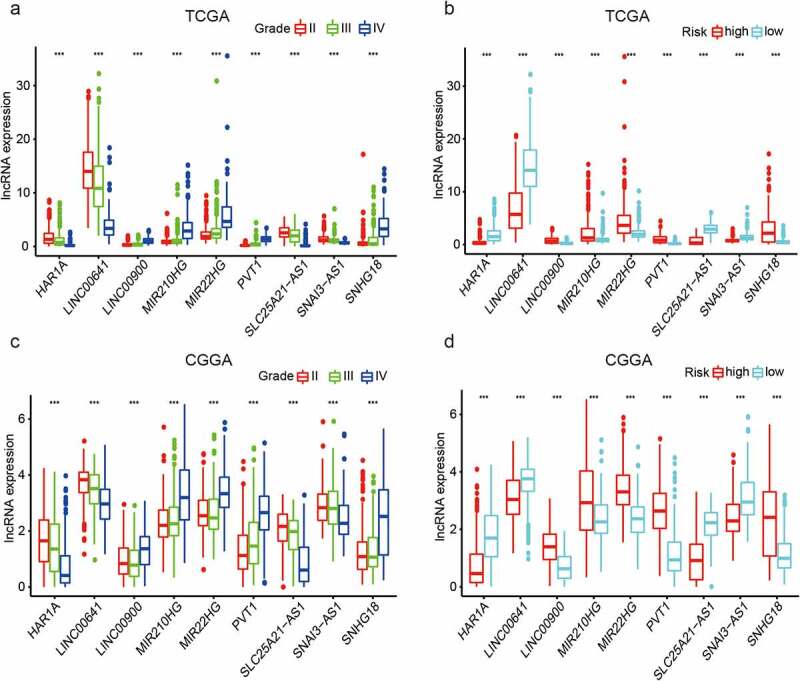
Expression of nine EMT-related lncRNAs according to tumor grade and risk group. ns P > 0.05, * P < 0.05, ** P < 0.01, and *** P < 0.001

We evaluated the expression of the nine EMT-related lncRNAs according to tumor grade in TCGA training set and the CGGA validation dataset. LINC00900, MIR210HG, MIR22HG, PVT1, and SNHG18 expression increased, whereas HAR1A, LINC00641, SLC25A21-AS1, and SNAI3-AS1 expression decreased with tumor grade (Figure 3a, c). LINC00900, MIR210HG, MIR22HG, PVT1, and SNHG18 levels were higher, whereas those of HAR1A, LINC00641 SLC25A21-AS1, and SNAI3-AS1 were lower in the high-risk group than in the low-risk group (Figure 3b, d). The results from the two datasets were consistent. We also examined the significance of these nine lncRNAs in terms of patient survival using TCGA dataset. Based on the expression level of each lncRNA, the survival time associated with each gene differed significantly between the high- and low-risk groups (Figure S1).

### Prognostic performance of risk scores and relationship with clinical status of glioma patients

**Figure 4.**
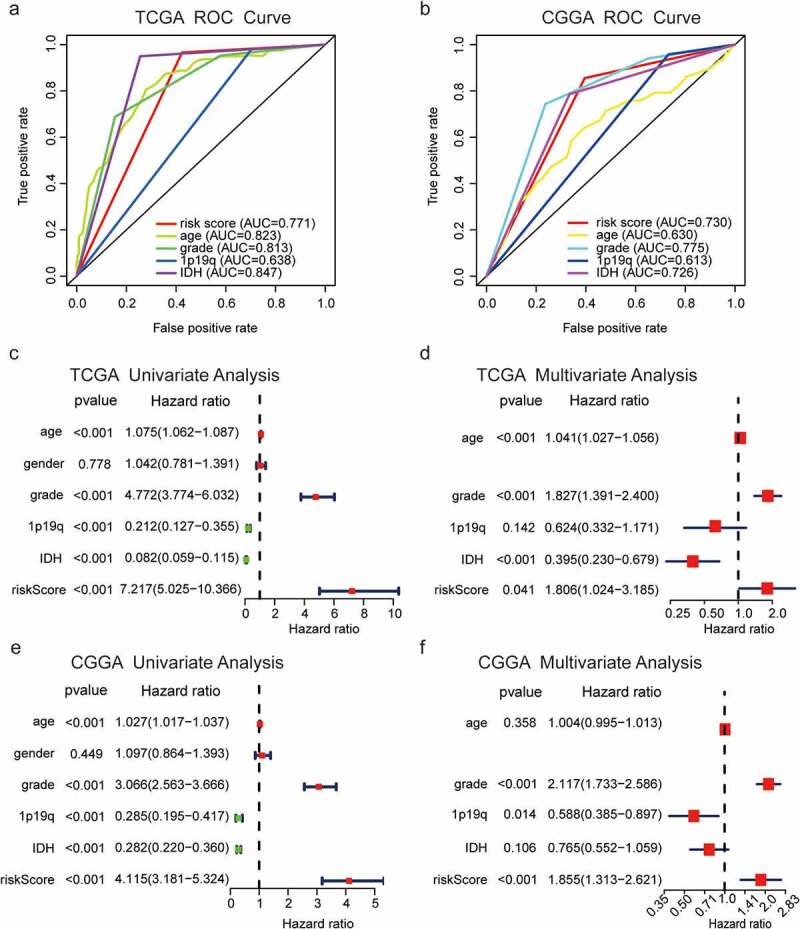
Predictive efficiency of the nine-EMT-related lncRNA signature. (a, b) Association between WHO grade, age, 1p19q codeletion status, IDH mutation status, and survival rate. (c–f) Univariate and multivariate Cox regression analyses of the association between clinicopathological factors (including risk score) and OS of patients in TCGA and the CGGA datasets

ROC curves of TCGA dataset showed that risk score (area under the ROC curve [AUC] = 0.771), age (AUC = 0.823), tumor grade (AUC = 0.813), 1p/19q codeletion status (AUC = 0.638), and IDH gene mutation status (AUC = 0.847) could accurately predict survival in patients with glioma (Figure 4a). These results were confirmed using the CGGA dataset (risk score, AUC = 0.730; age, AUC = 0.630; tumor grade, AUC = 0.775; 1p/19q codeletion status, AUC = 0.613; and IDH mutation status, AUC = 0.726; Figure 4b), demonstrating the reliability and effectiveness of the prognostic signature. Univariate and multivariate Cox regression analyses performed using TCGA dataset showed that risk score, age, WHO grade, and IDH mutation status were related to OS (Figure 4c, d), which was confirmed using the CGGA validation dataset (Figure 4e, f). These results suggested that the risk score derived from EMT-related lncRNAs was an independent factor that predicted the prognosis in patients with glioma (TCGA: P = 0.041, HR = 1.806; CGGA: P < 0.001, HR = 1.855; Figure 4c–f).

### PCA and functional annotation of EMT-related lncRNAs

**Figure 5.**
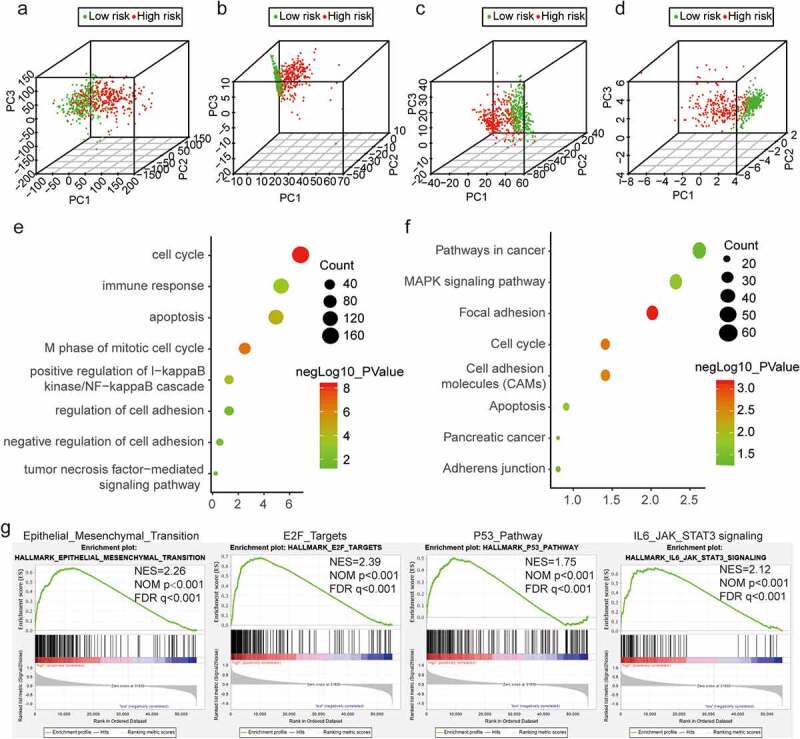
Distinct EMT status in patients with low-risk and high-risk glioma. (a) PCA between the low-risk and high-risk groups based on total gene expression profiles. (b) PCA between low-risk and high-risk groups based on EMT-related genes. (c, d) PCA between low-risk and high-risk groups based on all candidate EMT-related lncRNAs and the nine identified EMT-related lncRNAs. (e, f) GO and KEGG pathway enrichment analyses of DEGs in low-risk and high-risk groups. (g) Significant enrichment of EMT-associated functions in the high-risk group, as determined by GSEA

We performed PCA to investigate differences between the low-risk and high-risk groups based on EMT-related and overall gene expression profiles (Figure 5a–d). The low-risk and high-risk groups were distinguished based on EMT gene expression levels. We also found that EMT status was associated with a specific lncRNA signature. We performed GO and KEGG pathway analyses of the top 3,000 genes that were differentially expressed between the low-risk and high-risk groups and found that DEGs were enriched for the following GO terms: M phase of cell cycle, apoptosis, tumor necrosis factor-mediated signaling pathway, and regulation of cell adhesion. Additionally, KEGG pathway analysis revealed that focal adhesion, adherens junction, pathways in cancer, and cell adhesion molecules were significantly enriched (Figure 5e, f). Functional annotation by GSEA showed that DEGs between the two groups were enriched in EMT, E2F targets, the P53 pathway, and IL6/JAK/STAT3 signaling (Figure 5g). These results indicated that the two risk groups were strongly correlated with the malignancy of glioma cells.

### LncRNA-mediated ceRNA network and mechanisms of glioma tumorigenesis

**Figure 6.**
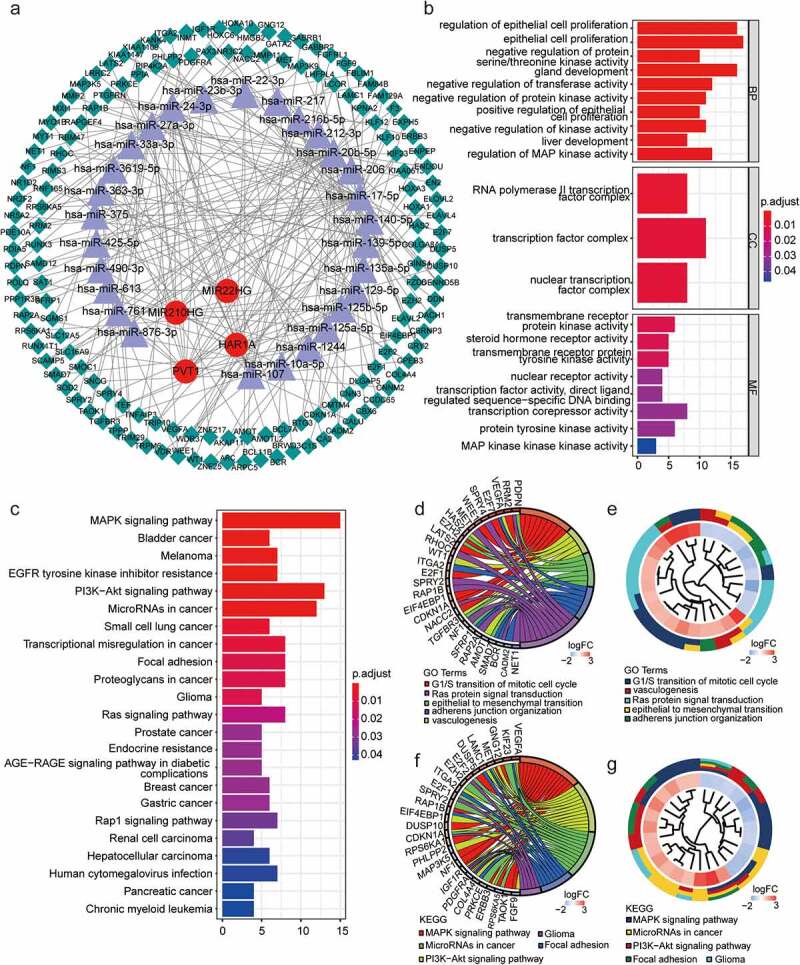
EMT-related ceRNA network and functional analysis of EMT-related lncRNAs. (a) Global view of the ceRNA network in glioma. Circles, triangles, and diamonds represent lncRNAs, miRNAs, and mRNAs, respectively. (b, c) GO and KEGG pathway enrichment analyses of mRNAs in the network. The horizontal axis shows the number of enriched genes, and the color intensity represents the corrected P value. (d, f) Genes in significant bioprocesses. (e, g) Cluster analysis of genes and pathways with related functions and enrichment of each functional pathway

To further investigate the regulatory role of lncRNAs in glioma, a lncRNA–miRNA–mRNA network was established by integrating expression profile data and regulatory relationships. We identified 220 EMT-related lncRNA–miRNA pairs comprising four lncRNAs and 28 miRNAs. MiRNA–mRNA pairs were predicted using the miRTarBase, TargetScan, and miRDB databases; all target mRNAs with matches in these databases were included in the analysis. In total, 145 target mRNAs (Table 2) were included in the co-expression network that combined lncRNA–miRNA and miRNA–mRNA pairs (Figure 6a). This ceRNA network revealed complex lncRNA–miRNA–mRNA interactions that could potentially contribute to EMT in glioma. GO and KEGG pathway enrichment analyses showed that the target genes of ceRNAs were mainly enriched for the following biological functions and pathways: vasculogenesis, adherens junction organization, Ras protein signal transduction, mitogen-activated protein kinase signaling, miRNAs in cancer, transcriptional misregulation in cancer, focal adhesion, and glioma (Figure 6b, c). We also identified genes of related functions and pathways and examined the enrichment of each functional pathway (Figure 6d–g). The results showed that the EMT-related ceRNA network played an important role in glioma progression.

**Table 2.** The EMT-related lncRNAs, miRNAs, and mRNAs included in the ceRNA network

The type of RNAs	Gene symbols
LncRNA	MIR22HG, HAR1A, MIR210HG, PVT1
miRNA	hsa-miR-23b-3p, hsa-miR-20b-5p, hsa-miR-363-3p, hsa-miR-17-5p hsa-miR-139-5p, hsa-miR-125b-5p, hsa-miR-27a-3p, hsa-miR-125a-5p hsa-miR-24-3p, hsa-miR-206, hsa-miR-140-5p, hsa-miR-129-5p, hsa-miR-107, hsa-miR-33a-3p, hsa-miR-1244, hsa-miR-10a-5p hsa-miR-216b-5p, hsa-miR-876-3p, hsa-miR-490-3p, hsa-miR-217 hsa-miR-425-5p, hsa-miR-135a-5p, hsa-miR-22-3p, hsa-miR-3619-5p hsa-miR-761, hsa-miR-212-3p, hsa-miR-375, hsa-miR-613
mRNA	TNFAIP3, KIF23, SLC12A5, LCOR, E2F2, MET, RHOC, PHLPP2, TRIP10, PIP4K2A, GINS4, RRM2, MMP11, KPNA2, ZNF217, SGMS1, SAMD12, SFRP1, PDGFRA, CDKN1A, RBM47, ELAVL2, RUNX3, PRKCE, BTG3 DLGAP5, SLC16A9, PAX3, SAT1, BCL7A, GOLGA8A, RAP1B, LAMC1, WDR37, KLF10, EIF4EBP1, HOXA3, VEGFA, CMTM4, LHFPL4, RAPGEF4, NACC2, FZD6, RPS6KA5, KLF12, AKAP11, PPIA, AMOT, MAP3K9, E2F1, KIAA0513, AMOTL2, HOXA1, TEF, CNN3, RNF165, SPRY4, SNCG, RIMS3, EZH2, HAS2, PTGFRN, NR3C2, SMOC1, SCAMP5, WEE1, DUSP5, TGFBR3, CADM2, GNG12, DUSP10, TRPM6,IGF1R, NF1, HOXC6, CBX6, ELOVL2, GABBR2, MYT1, RPS6KA1, CPEB3, SPRY2, ERBB3, TRIM29, CALU, ARC, INMT, CCDC65, POLQ, SOD2, EXPH5, WT1, NR2F2, NET1, CA2, CRY2, ITGA2, KANK4, COL4A4, MMP2, FAM129A, PDIA5, LRRC2, MAP3K5, ENDOU, PDPN, CSRNP3, ZNF25, MXI1, FAM84B, TPPP, PPP1R3B, FGF9, DDN, GATA2, EN2, MYO1B, ARPC5, F3, BCL11B, FBLIM1, BCR, KIAA1147, VDR, NR5A2, BRWD3, DENND5B, GABRB1, TAOK1, C1S, CNNM2, FGFRL1, RUNX1T1, E2F7, SMAD7, ELAVL4, NR1D2, DACH1, ENPEP, KIAA1109, HMGB2, HOXA10, LATS2, PDE10A, RAP2A

### Establishment of the TFs‐LncRNAs network, and the expression of HAR1A and PVT1 is associated with 6 types of infiltrating immune cell in gliomas

**Figure 7.**
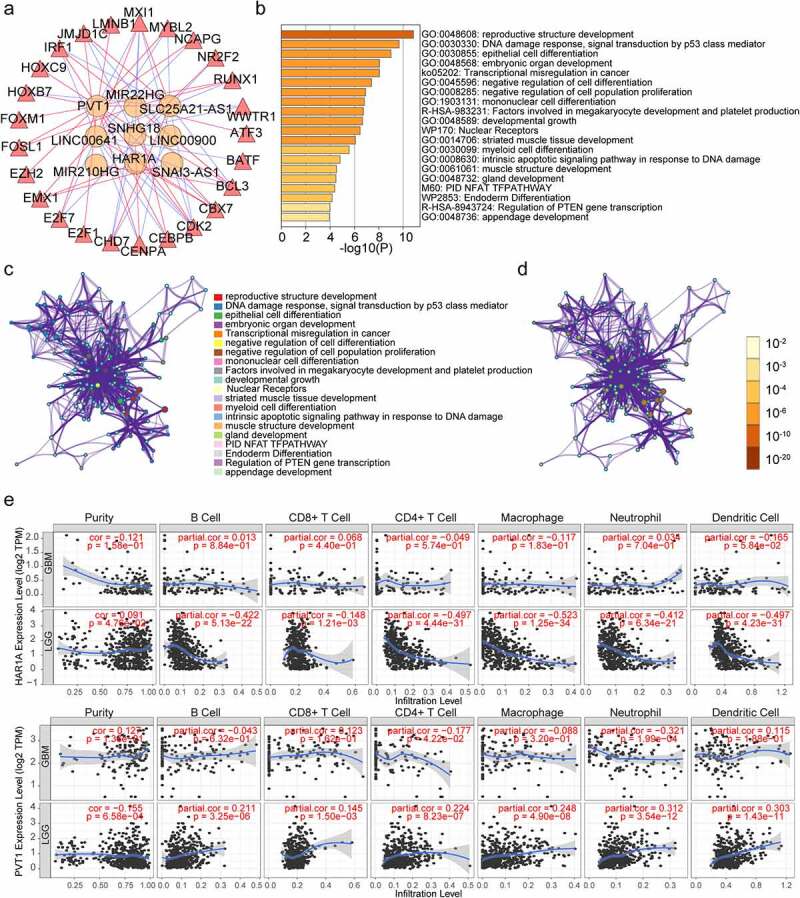
Establishment of TFs‐LncRNAs regulatory networks and associations between the expression of EMT-related lncRNAs and immune infiltration in glioma. (a)The network based on potential regulatory mechanisms between TFs and the nine EMT-related lncRNAs. (b) Heatmap of enriched terms across the 25 TFs, colored according to p-value. Network of enriched terms colored according to (c) cluster ID (nodes with the same cluster ID are typically close to each other) and (d) p-value (terms with more genes tend to have higher p-values). (e) associations between the expression of EMT-related lncRNAs and immune infiltration in glioma

To further study the transcriptional regulation of these LncRNAs, we sought to some TFs which could regulate our LncRNAs expression. Out of 318 TFs, 42 TFs were found differentially expressed in gliomas (FDR<0.05, logFC>1). Then we calculated correlation score between 42 TFs and the nine LncRNAs, a correlation score more than 0.4 and p < 0 .001 were set as the cut‐off values. Finally, we selected 25 TFs to construct the TFs‐LncRNAs network (Figure 7a). Then, we perform functional enrichment analysis of these 25 TFs in the Metascape online tool, we found that these TFs were enriched in DNA damage response, signal transduction by p53 class mediator, epithelial cell differentiation, Transcriptional misregulation in cancer, and negative regulation of cell population proliferation (Figure 7b–d). These data may provide us some clues for verifying the potential functions of these EMT-related lncRNAs in gliomas. We explored the association between HAR1A and PVT1 (other LncRNA data missing) expression and immune infiltration levels of 6 types of immune cells, namely, B cells, CD8 + T cells, CD4 + T cells, macrophages, neutrophils, and dendritic cells in GBM and LGG using the TIMER dataset. These analyses revealed that low expression levels of HAR1A could critically decrease immune infiltrating levels of B cells (R = －0.422, P = 5.13e-22), CD8 + T cells (R = －0.418, P = 1.21e-03), CD4+ cells (R = －0.497, P = 4.44e-31), macrophages (R = －0.523, P = 1.25e-34), neutrophils (R = －0.412, P = 6.34e-21), dendritic cells (R = －0.497, P = 4.23e-31) in LGG, high expression levels of PVT1 could critically increase immune infiltrating levels of B cells (R = 0.211, P = 3.25e-06), CD8 + T cells (R = 0.145, P = 1.50e-03), CD4+ cells (R = 0.224, P = 8.23e-7), macrophages (R = 0.248, P = 4.90e-8), neutrophils (R = 0.312, P = 3.54e-12), dendritic cells (R = 0.303, P = 1.43e-11) in LGG. The expression of HAR1A and PVT1 were have little relationship with infiltration levels of the 6 types of immune cells in GBM (Figure 7e).

### mRNA and protein expression levels of the nine selected lncRNAs in glioma

**Figure 8.**
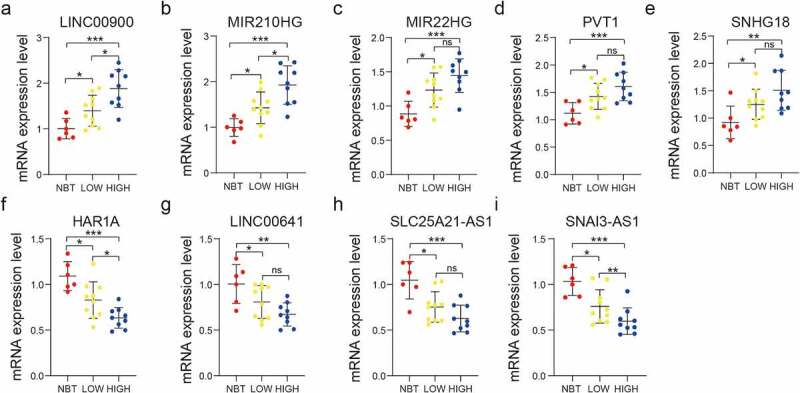
Validation of the bioinformatics results using RT-qPCR assay. Comparison of LINC00900 (a), MIR210HG (b), MIR22HG (c), PVT1 (d), SNHG18 (e), HAR1A (f), LINC00641(g), SLC25A21-AS1 (h), and SNAI3-AS1 (i) mRNA expression levels in NBT, LGG, and GBM tissues. ns P > 0.05, * P < 0.05, ** P < 0.01, and *** P < 0.001

We performed RT-qPCR assays to validate the bioinformatics results. These assays showed that the nine selected lncRNAs (LINC00900, MIR210HG, MIR22HG, PVT1, SNHG18, HAR1A, LINC00641, SLC25A21-AS1, and SNAI3-AS1) were expressed to different extents in NBT, LGG, and GBM tissues at the mRNA level (Figure 8a–i), in accordance with the bioinformatics results.

## Discussion

In this study, we identified a nine-lncRNA signature that could distinguish between low-risk and high-risk patients with glioma based on the median risk score in TCGA. These nine EMT-related lncRNAs have prognostic significance in gliomas; patients in the low-risk group had a longer OS than those in the high-risk group. Deletion of chromosomal arms 1p and/or 19q and IDH mutations are considered useful biomarkers in glioma [2,28,29]; We evaluated the risk scores of IDH and 1p/19q codeletion status in TCGA dataset and found that both were related to OS. The results were confirmed using the CGGA dataset, indicating that the nine-lncRNA signature is reliable and effective for predicting prognosis. We also established lncRNA–miRNA–mRNA ceRNA network to predict the regulatory relationships of the nine lncRNAs; and TFs‐LncRNAs network to study the transcriptional regulation of these LncRNAs. Functional enrichment analysis of downstream target genes revealed that these lncRNAs are involved in tumorigenesis and cancer progression.

EMT is an intricate genetic procedure that can be utilized by both normal and tumor epithelial cells to enable them to separate from neighboring cells and migrate [30], influencing invasion, cancer progression, and the therapeutic resistance properties of cancer cells [31,32]. Previous studies have indicated that the Snail [33,34], ZEB [35,36], Twist [37] families and some other signaling pathways genes [38–40] are master regulators of EMT progression. Since we set certain standards in the screening process, the lnc RNAs included in the model building have no direct role or connection with these genes, but we consider that they may be a synergistic role in the process of EMT. Increasing evidence shows that lncRNAs epigenetically orchestrate EMT, broadening our understanding of the molecular mechanisms of lncRNA-mediated cancer initiation and malignancy progression [6]. LncRNAs play a critical role in diverse cellular processes in both normal and disease states [12,41]. Specifically, lncRNAs promote or act as decoys to repress transcription or function as epigenetic regulators or scaffolds that interact with various proteins to form ribonucleoprotein complexes [42,43]. LncRNAs, including p53, nuclear factor κB, phosphatidylinositol 3-kinase/protein kinase B, and Notch are involved in multiple signaling pathways and have been linked to EMT. Among the nine EMT-related lncRNAs identified in this study, LINC00900, MIR210HG, MIR22HG, PVT1, and SNHG18 were positively correlated with glioma malignancy, whereas HAR1A, LINC00641, SLC25A21-AS1, and SNAI3-AS1 were negatively correlated with glioma malignancy. PVT1 is upregulated in various carcinomas, and its overexpression is associated with poor survival in patients [44]. PVT1 functions as a ceRNA to protect mRNAs from miRNA-mediated repression and is associated with the development of resistance to common chemotherapeutic agents [45,46]. MiR210HG sponges miR-1226-3p to facilitate breast cancer cell invasion and metastasis and EMT via regulation of mucin-1 c. Further, MIR210HG upregulation has been shown to be associated with advanced International Federation of Gynecology and Obstetrics stage, metastasis, and poor prognosis in cervical cancer [47,48]. SNHG18 promotes glioma resistance to radiotherapy by repressing semaphorin 5A [49]. LINC00641 expression levels are decreased in breast cancer tissue, which is negatively correlated with tumor size, lymph node metastasis, and clinical stage [50]. SNAI3-AS1 can bind to up-frameshift protein 1, regulate Smad7 expression, and activate the TGF-β/Smad pathway to induce EMT in hepatocellular carcinoma [51]. It also acts as an important factor in the ceRNA network in adipose tissue of patients with type 2 diabetes [52]. Therefore, the close relationship between lncRNAs and EMT that we identified and the important role of lncRNAs in the ceRNA network has been further confirmed in other tumors or diseases. Although the association between some of the identified lncRNAs and EMT has not been reported previously, our results suggest that they may be directly or indirectly involved in this process.

This study has some limitations. First, our analyses were based on publicly available datasets and require confirmation in other patient populations. Moreover, more experiments should be performed to validate the roles of the identified lncRNAs, their targets, and the lncRNA–miRNA–mRNA ceRNA network in glioma. In conclusion, the nine-EMT-related lncRNA signature identified in this study has a prognostic value in gliomas. The ceRNA network provides insight into the molecular basis of glioma progression and potential therapeutic targets for its treatment.

## Supplementary Material

Supplemental Material
