# Supplementary material for: Identification of an epithelial-mesenchymal transition related long non-coding RNA (LncRNA) signature in Glioma
Source: Bioengineered. 2021 Jul 21;12(1):4016–31. doi: 10.1080/21655979.2021.1951927 (PMC8806607; doi:10.1080/21655979.2021.1951927)
Supplement: Supplemental Material [file KBIE_A_1951927_SM1373.zip › supplementary/Table S3.docx]

**Table S3.** Primer sequences used for Realtime PCR analysis.

| **Gene symbol** | **Gen Bank Accession no.** | **Primer set sequence (5’->3’)** | **Amplicon size (bp)** |
| --- | --- | --- | --- |
| *LINC00900* | NR_034148.1 | Forward:  AACGCTGACACTGATGACCC  Reverse:  TTGAGGGGAGGGGTGAAAGA | 176 |
| *MIR210HG* | NR_038262.1 | Forward:  TAACTTACTGCCAGACGGCG Reverse:  CAGAGCCAGGCTGTATCGAG | 152 |
| *MIR22HG* | NR_028502.1 | Forward:  AGCCCCTTGGTGGGATTCTA  Reverse:  ACAACCCACCTCCCCTACTT | 136 |
| *PVT1* | NR_003367.3 | Forward:  GAGCTGCGAGCAAAGATGTG  Reverse:  AAAAGATCAGGCTGGAGGGC | 127 |
| *SNHG18* | NR_045196.2 | Forward:  TGCCATCTCAGACCAGAGGA  Reverse:  AAAGACCACAGATACCCGGC | 124 |
| *HAR1A* | NR_003244.2 | Forward:  TCAGCTGAAATGATGGGCGT Reverse:  CATCGCGGAAAACGGGATTC | 117 |
| *LINC00641* | NR_038970.1 | Forward:  AGCAGGAGTAGCCCTTGGTA  Reverse:  ATTCCCAGGTGAGAGGGGAT | 130 |
| *SLC25A21-AS1* | NR_033240.1 | Forward:  ATCAGGAGCCTCCGGTAGTT  Reverse:  TGAACCTCACTGTGCTCTGG | 138 |
| *SNAI3-AS1* | NR_024402.2 | Forward:  GGTCTGGCAAGAATGCCTCT  Reverse:  GCTCCCTGGCAGAGTTCAAT | 208 |
