# Supplementary figures and images for: Identification of an epithelial-mesenchymal transition related long non-coding RNA (LncRNA) signature in Glioma
Source: Bioengineered. 2021 Jul 21;12(1):4016–31. doi: 10.1080/21655979.2021.1951927 (PMC8806607; doi:10.1080/21655979.2021.1951927)

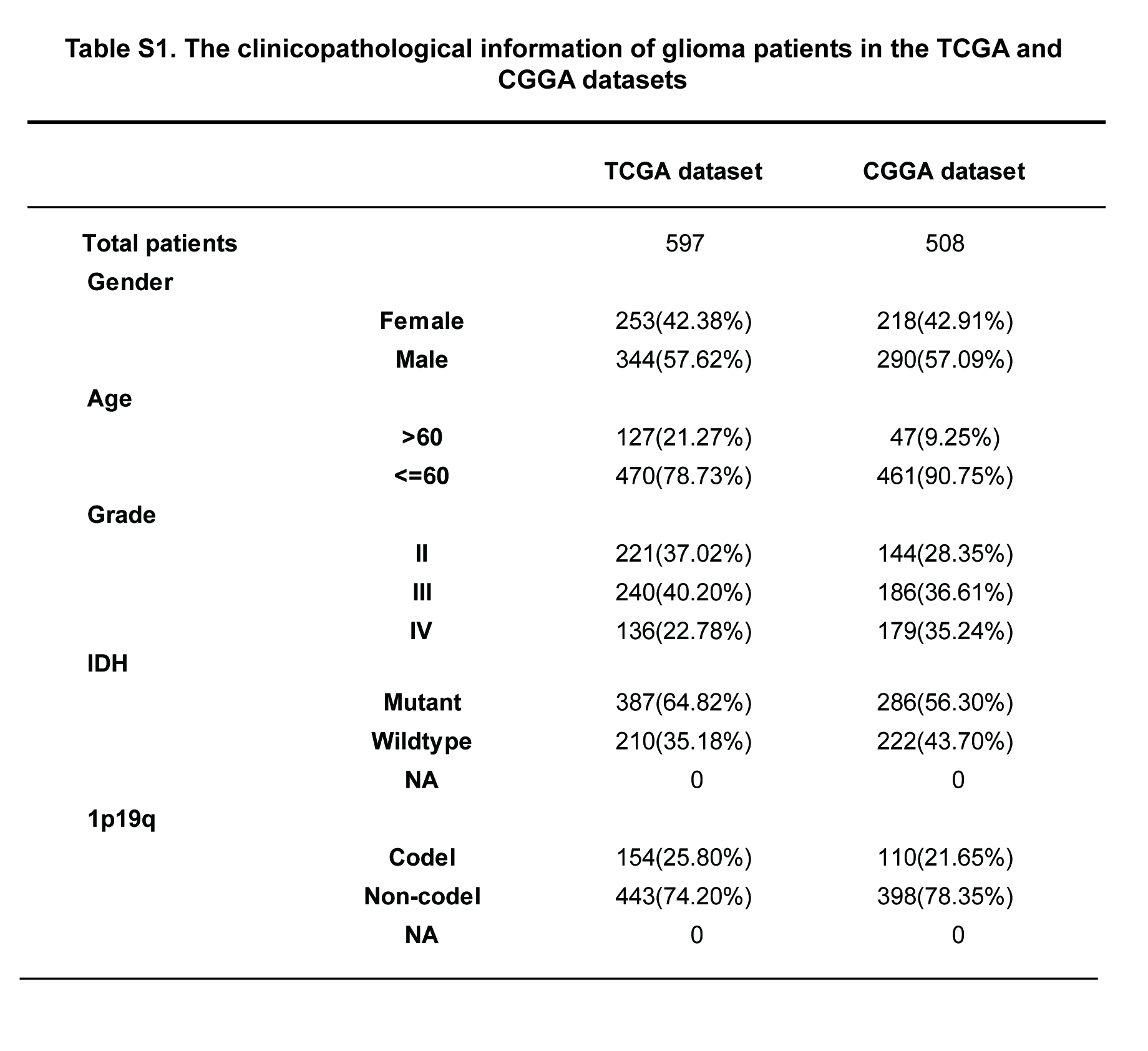

Supplement: Supplemental Material [file KBIE_A_1951927_SM1373.zip › supplementary/table S1.tif]

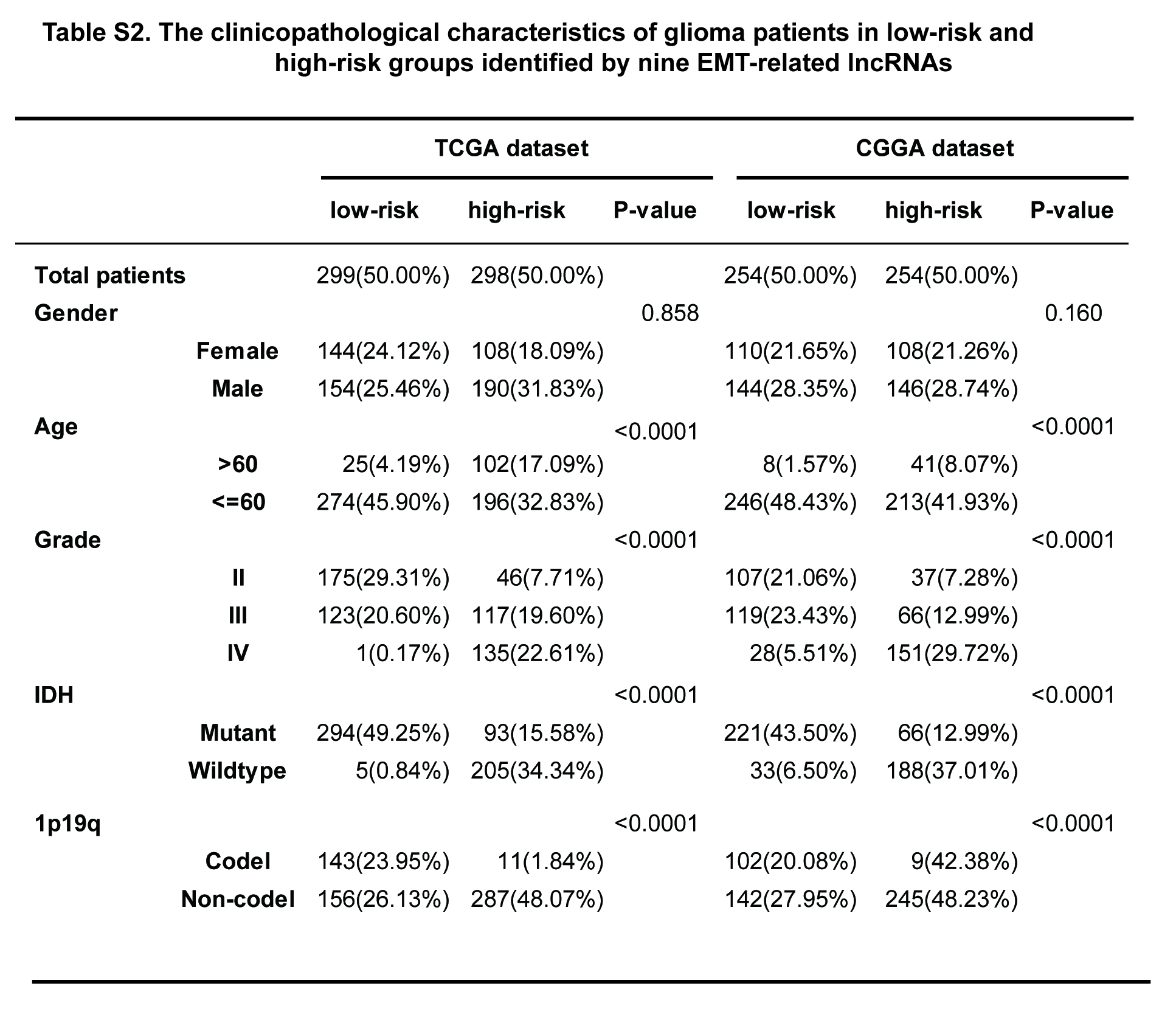

Supplement: Supplemental Material [file KBIE_A_1951927_SM1373.zip › supplementary/table S2.tif]
